# The association between *phosphatase and tensin homolog* hypermethylation and patients with breast cancer, a meta-analysis and literature review

**DOI:** 10.1038/srep32723

**Published:** 2016-09-13

**Authors:** Yi-Min Lu, Feng Cheng, Li-Song Teng

**Affiliations:** 1 Department of Oncological Surgery, The First Affiliated Hospital, School of Medicine, Zhejiang University, 79 Qingchun Road, Hangzhou, Zhejiang, 310003, P. R. China; 2 Department of Thyroid-Breast Surgery, The Central Hospital of Lishui City, 289 Kuocang Road, Lishui, Zhejiang, 323000, P. R. China

## Abstract

The Phosphatase and tensin homolog (PTEN) protein is a negative regulator of the Akt pathway, leading to suppression of apoptois and increased cell survival. Its role as a tumor-suppressor gene has been adequately substantiated, and *PTEN* hypermethylation has been demonstrated in familial and sporadic cancers. However, the association and clinical significance between *PTEN* hypermethylation and breast cancer remains unclear. In this study, we systematically reviewed studies of *PTEN* hypermethylation and breast cancer and quantify the association between *PTEN* hypermethylation and breast cancer using meta-analysis methods. The pooled OR, 22.30, 95% confidential intervals, CI = 1.98–251.51, *P* = 0.01, which demonstrates that loss of *PTEN* expression by hypermethylation plays a critical role in the early tumorigenesis of ductal carcinoma in situ (DCIS). In addition, *PTEN* hypermethylation also is detected in invasive ductal carcinomas (IDCs) and is significantly higher than in normal controls, OR = 23.32, 95% CI = 10.43–52.13, *P* *<* 0.00001. Further analysis did not show significant correlation between *PTEN* hypermethylation and the progression of breast cancer, estrogen receptor (ER), progesterone receptor (PgR), as well as HER2 status. These results indicate the *PTEN* hypermethylation is significantly associated with both DCIS and IDCs. The detection of *PTEN* hypermethylation could be an early tumorigenesis marker for breast cancer patients.

Breast cancer is the most common cancer among women worldwide. Breast cancer develops as a stepwise accumulation of genetic changes (point mutations, deletions, and gene amplifications) leading to oncogene activation or tumor suppressor gene inactivation^1^. In addition to genetic alterations, breast cancer progression is also controlled by epigenetic modifications such as DNA methylation, histone acetylation and nucleosomal remodeling^2^. Phosphatase and tensin homolog (PTEN) is a protein encoded by the tumor suppressor gene *PTEN*. It plays a critical role in cell growth, proliferation and differentiation^3^ and is involved in DNA repair and corresponding signaling in breast cancer^4^. First, PTEN is important in breast cancer initiating cells (CICs) survival. Knockdown of PTEN expression causes increases in normal and malignant mammary stem/progenitor cells, which was regulated by increased AKT/GSK-3beta/Wnt/beta-catenin pathway^5^,^6^. The resistance of breast cancers to herceptin (trastuzumab) and other chemotherapies are therefore related to loss of PTEN expression^6^,^7^,^8^,^9^,^10^. Second, multiple mechanisms of PTEN inactivation applies in breast cancer. Reduced expression of PTEN in breast cancer may result from *PTEN* gene as mutation, deletion and methylation^9^. The role of PTEN protein in cancers has been widely studied. For instance, a couple of meta-analyses demonstrated that PTEN was associated with poor survival in gastric, colorectal, as well as endometrial and prostate cancers^11^,^12^. A prognostic value of PTEN in breast cancer has also recently been investigated using meta-analysis. They solidified that PTEN inactivation is associated with unfavorable overall survival and disease-free survival^13^. However the systemic review on evaluation of *PTEN* methylation in breast cancer patients has not been documented. In the present study we performed a meta-analysis and systemic review on the relationship of *PTEN* hypermethylation and clinical parameters of human breast cancer to fill the gap of breast cancer research.

## Material and Methods

### Search Method

We systematically searched Embase, Pubmed and ISI web of knowledge to select studies from June 1, 1996 through April 1, 2016. Keywords used to search relevant publications included: “breast cancer”, “breast tumor”, and “breast carcinoma”, “methylation”, “hypermethylation”, “epigenetic changes”, “ phosphatase and tensin honolog”, and “*PTEN*”. We also scrutinized the reference lists of the relevant reviews and eligible articles for additional eligible articles. No restrictions were placed on language and publication status.

### Selection criteria

All searched data were retrieved. The titles and abstracts of all searched records were scrutinized to judge their relevance and eligibility. After excluding of duplicates and publications with non-extractable data, the remaining papers were screened in the full text version for in- and exclusion criteria. The most complete investigations was included if the same patient populations were published in different resources. Cohort studies that fulfills the following criteria were considered eligible: (1) *PTEN* methylation examined in breast cancer patients, (2) *PTEN* methylation determined by polymerase chain reaction (PCR), (3) researches revealed the relationship between *PTEN* methylation status and breast cancer clinicopathological parameters and the outcomes, (4) studies which provided sufficient data to calculate Odds ratio (OR) and/or hazard ratio (HR) about overall survival (OS) and 95% confidence interval (CI). The exclusion criteria: (1) all the descriptive studies including reviews, letters, case studies, editorials, and conference abstracts, (2) all *in vitro* and *in vivo* laboratory works.

### Data extraction and methodological assessment

Three authors independently completed the data extraction from eligible studies by evaluating all of records that meet the inclusion and exclusion criteria. The following data were extracted from each study: (1) bibliographic data including the first author name, year of publication, and country; (2) data on clinicopathological characteristics including number of cases, sample source, age, state of disease, histology, ER status, PR status and HER2 status; (3) methylation detection methods, methylation rate, and/or expression, and follow up. Data for study characteristics were summarized in a table format. Investigation heterogeneity was evaluated to determine whether or not the data of the various studies could be analyzed for a meta-analysis.

Three investigators read through each publication independently for the methodological evaluation of the studies, and assessed and scored them according to REMARK guidelines and ELCWP quality scale^14^,^15^. Then they provided the quality scores, compared them, and then reached a consensus value for each item if disagreements existed between the investigators.

### Statistical analysis

Analysis was performed using the STATA 12.0 (Stata Corporation, TX, USA) and Review Manager 5.2 (Cochrane Collaboration, Oxford, UK). The pooled rate of *PTEN* hypermethylation and 95% confidence intervals (95% CI) from different studies were combined to obtain a summary estimate. A summary Odds Ratio was estimated and compared for the inter-relationship between PTEN and each tumor factors/characteristics. Heterogeneity among studies was examined with Cochran’s Q test^16^ and the *I*^*2*^ statistic^17^,^18^. A fixed effect model was used for meta-analyses when heterogeneity was not an issue (*I*^*2*^ values <50% or *P* value ≤0.10), while a random-effects model was used to pool data and attempt to identify potential sources of heterogeneity based on subgroup analyses, if there was substantial heterogeneity (*I*^*2*^ values ≥50%). *P* values tailed less than 0.05 were considered statistically significant.

The analysis of meta-regression and publication bias was performed using STATA version 10.0. The possibility of publication bias was determined by using Begg’s funnel plot and Egger’s regression test^19^. For statistical heterogeneity, we determined the reasons using meta-regression, subgroup analysis and sensitivity analysis.

### Patient survival analysis

An online database^20^ was used to assess relevance of PTEN expression to relapse free survival. The database was established using gene expression data and survival information of 3,557 patients downloaded from Gene Expression Omnibus (GEO) (Affymetrix HGU133A and HGU133+2 microarrays). Briefly, PTEN gene was entered into the database (http://kmplot.com/breast/) to obtain Kaplan-Meier survival plots where the number-at-risk is indicated below the main plot. Hazard ratio (and 95% confidence intervals) and logrank P were calculated and displayed on the webpage.

Another online tool we used is OncoLnc, a tool for interactively exploring survival correlations, and for downloading clinical data coupled to expression data for mRNAs, miRNAs, or long noncoding RNAs (lncRNAs)^21^. OncoLnc contains survival data for 8,647 patients from 21 cancer studies performed by The Cancer Genome Atlas (TCGA), along with RNA-SEQ expression for mRNAs and miRNAs from TCGA, and lncRNA expression from MiTranscriptome beta. This dataset contains 995 breast invasive cancer (BRCA) patients, OncoLnc is available at http://www.oncolnc.org. The multivariate cox regressions were performed followed by a Kaplan-Meier analysis for BRCA.

## Results

**Figure 1:**
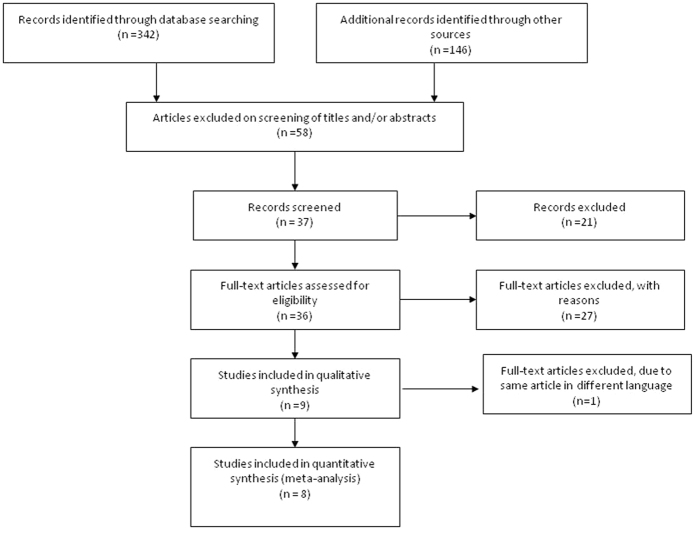
Flow chart of include studies.

Initially, 488 articles were identified by the literature search. Among these references, 9 studies were considered potentially eligible, and 8 studies published between 2004 to 2015 were finally included in this meta-analysis. The flow chart of study is shown in Fig. 1. The articles were excluded due to reviews*, in vitro* and *in vivo* laboratory studies.

### Included study characteristics

The basic characteristics of included studies were summarized in Table 1. Eight studies were conducted in United States, Norway, Spain, China, India and Iran. The sample sizes ranged from 28 to 199, with a total of 927 breast cancer patients and 588 corresponding controls. PTEN status was consistently examined by methylation specific PCR (MSP) using breast cancer tissues across the included studies.

### *PTEN* hypermethylation and clinicopathological characteristics

#### The *PTEN* hypermethylation in ductal carcinoma in situ (DCIS) and invasive ductal carcinomas (IDCs)

**Figure 2:**
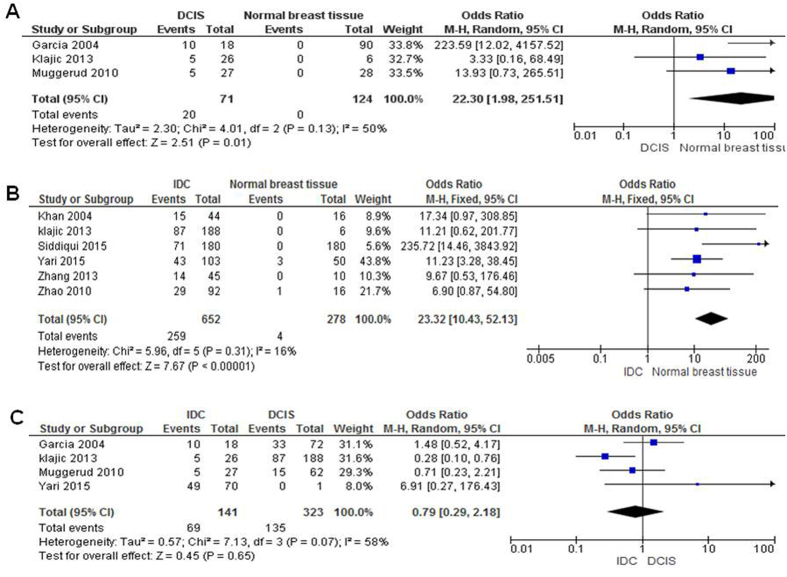
The loss of *PTEN* expression through hypermethylation in ductal carcinoma in situ (DCIS) and invasive ductal carcinomas (IDCs). (**A**) The patients with DCIS showed high level of *PTEN* hypermethylation compared to normal individuals. The pooled OR from 3 studies including 71 of DCIS and 124 of normal breast tissues are shown (odds ratios, OR = 22.30, 95% confidential intervals, CI = 1.98–251.51, *P* = 0.01), which demonstrates that loss of *PTEN* expression by hypermethylation plays a critical role in the early tumorigenesis of DCIS. (**B**) *PTEN* hypermethylation also is detected in IDC and is significantly higher than in normal controls (OR = 23.32, 95% CI = 10.43–52.13, *P* *<* 0.00001). (**C**) There is no significant difference between *PTEN* hypermethylation between individuals with IDC and with DCIS, OR = 0.79 95% CI = 0.29–2.18, *P* = 0.65.

The patients with DCIS showed high level of *PTEN* hypermethylation compared to normal individuals. The pooled OR from 3 studies including 71 of DCIS and 124 of normal breast tissues are shown in Fig. 2A (odds ratios, OR = 22.30, 95% confidential intervals, CI = 1.98–251.51, *P* = 0.01), which demonstrates that loss of *PTEN* expression by hypermethylation plays a critical role in the early tumorigenesis of DCIS. In addition, *PTEN* hypermethylation also is detected in IDCs and is significantly higher than in normal controls (OR = 23.32, 95% CI = 10.43–52.13, *P* *<* 0.00001), as shown in Fig. 2B. There is no significant difference between *PTEN* hypermethylation between individuals with IDCs and with DCIS, OR = 0.79 95% CI = 0.29–2.18, *P* = 0.65 (Fig. 2C). The blue rectangle in each figure showed the individual OR where as the diamond showed the total OR.

#### *PTEN* hypermethylation in the progression of breast cancer

**Figure 3:**
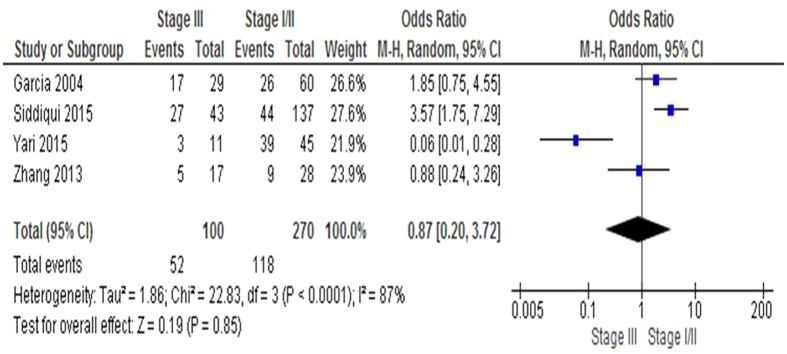
*PTEN* hypermethylation in the progression of breast cancer. 540 breast cancer patients pooled in 4 studies to evaluate the role of inactivation of *PTEN via* hypermethylation in the progression of breast cancer. The aberrant *PTEN* hypermethylation is not significantly higher in advanced stage of breast cancer (III) than that in early staged breast cancer (I & II), OR = 0.87, 95% CI = 0.20–3.72, *P* = 0.85. These results indicate that the inactivation of *PTEN* gene by hypermethylation may not play an important role in breast cancer progression from initial stage to advanced stage.

We then determined 540 breast cancer patients pooled in 4 studies to evaluate the role of inactivation of *PTEN via* hypermethylation in the progression of breast cancer. In Fig. 3, aberrant *PTEN* hypermethylation is not significantly higher in advanced stage of breast cancer (III) than that in early staged breast cancer (I & II), OR = 0.87, 95% CI = 0.20–3.72, *P* = 0.85. These results indicate that the inactivation of *PTEN* gene by hypermethylation may not play an important role in breast cancer progression from initial stage to advanced stage. The blue rectangle in each figure showed the individual OR where as the diamond showed the total OR.

#### Correlation with *PTEN* hypermethylation and hormone receptor status and HER2 status

**Figure 4:**
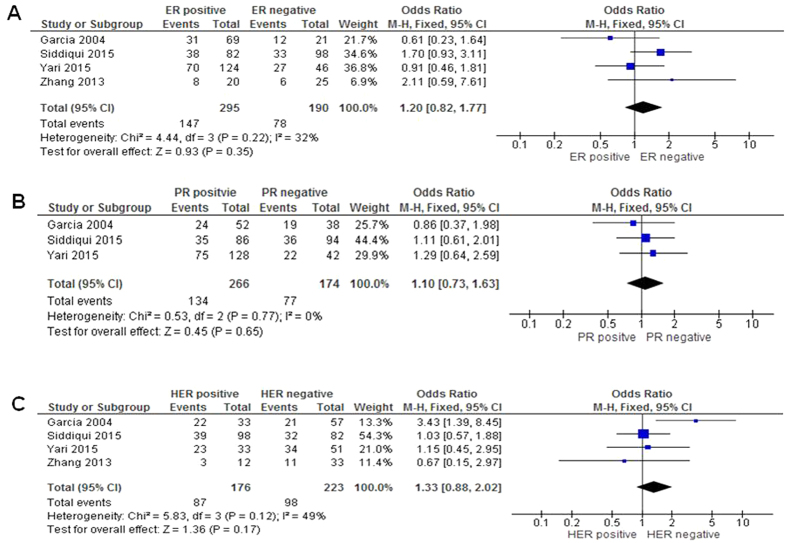
Correlation with *PTEN* hypermethylation and hormone receptor status and HER2 status. (**A**) No significant association with estrogen receptor (ER) status was observed for *PTEN* methylaltion, OR = 1.20 with 95%CI: 0.82–1.77 (*P* = 0.35). (**B**) No correlation with *PTEN* hypermethylation and PR. PR receptor status were not associated with *PTEN* methylation either OR = 1.10 95% CI = 0.73–1.63, *P* = 0.65. (**C**) No correlation with *PTEN* hypermethylation and HER2. HER2 receptor status were not associated with *PTEN* methylation either OR = 1.33 95% CI = 0.88–2.02, *P* = 0.17 respectively.

No significant association with estrogen receptor (ER) status was observed for *PTEN* methylaltion, OR = 1.20 with 95%CI: 0.82–1.77 (*P* = 0.35, Fig. 4A). PgR and HER2 status were not associated with *PTEN* methylation either OR = 1.10 95% CI = 0.73–1.63, *P* = 0.65; OR = 1.33 95% CI = 0.88–2.02, *P* = 0.17 respectively (Fig. 4B,C). The blue rectangle in each figure showed the individual OR where as the diamond showed the total OR.

#### The role of PTEN in breast cancer patient survival

**Figure 5:**
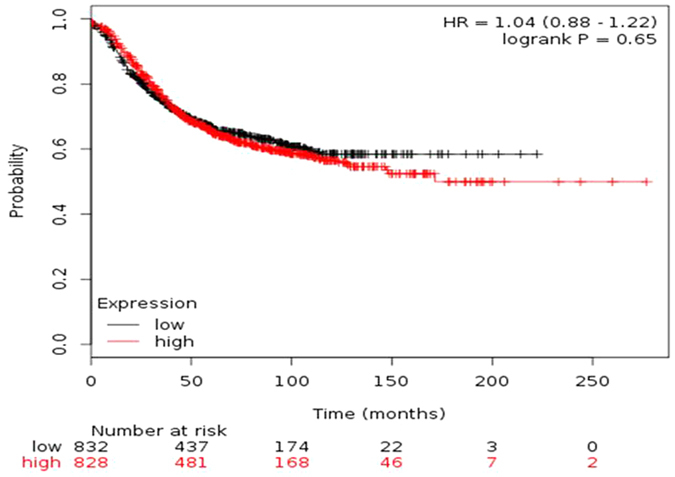
*PTEN* mRNA does not correlate with disease-free survival in breast cancer patients. This assessment of clinical relevance was performed in a patient survival analysis using an online database containing the expression of 54,675 genes and 20-year survival information of 4142 patients^20^, including survival information of 3557 breast cancer patients (http://www.kmplot.com/analysis/). PTEN downregulation was not found to correlate with shorter relapse free survival (RFS) for all breast cancer patients followed for 20 years (Fig. 5, hazardous ratio 1.04, p = 0.65).

This assessment of clinical relevance was performed in a patient survival analysis using an online database containing the expression of 54,675 genes and 20-year survival information of 4142 patients^20^, including survival information of 3557 breast cancer patients (http://www.kmplot.com/analysis/). PTEN downregulation was not found to correlate with shorter relapse free survival (RFS) for all breast cancer patients followed for 20 years (Fig. 5, hazardous ratio 1.04, p = 0.65).

**Figure 6:**
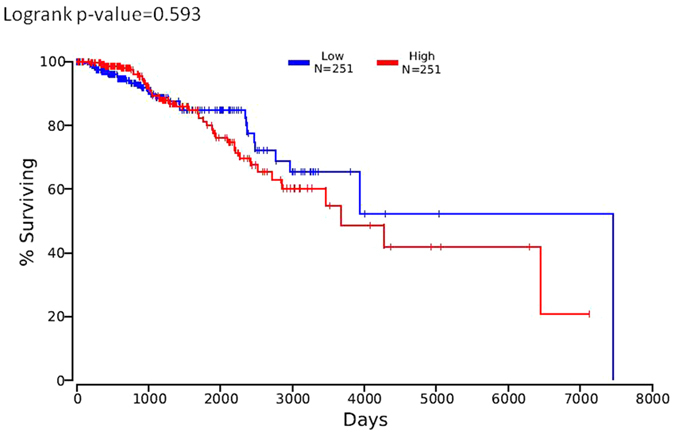
*PTEN* mRNA does not correlate with disease-free survival in breast invasive cancer patients (BRCA). By using the tool of OncoLnc to link TCGA survival data of breast invasive cancer (BRCA) patients to mRNAs, patients were split into non-overlapping upper and lower slices, namely upper 25 percent and lower 25 percent (Fig. 6). Similarly low expression of PTEN was not correlated with disease free survival (logrank p = 0.593).

By using the tool of OncoLnc to link TCGA survival data of breast invasive cancer (BRCA) patients to mRNAs, patients were split into non-overlapping upper and lower slices, namely upper 25 percent and lower 25 percent (Fig. 6). Similarly low expression of PTEN was not correlated with disease free survival (logrank p = 0.593).

#### Publication bias and sensitivity analyses

**Figure 7:**
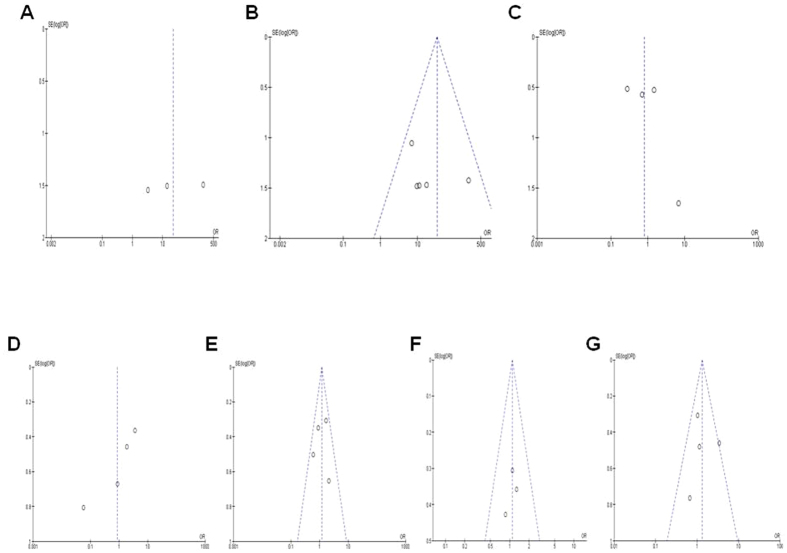
Publication bias and sensitivity analyses. In the current meta-analysis of *PTEN* hypermethylation and clinicopathological features, the publication biases were evaluated by the funnel plots. The publication biases were ruled out by the symmetric funnel plot. The funnel plots were largely symmetric suggesting there were no publication biases in the meta-analysis of *PTEN* hypermethylation and breast cancer clinicopathological features. The funnel plot from 3 studies comparing 91 ductal carcinoma in situ (DCIS) and 124 normal breast tissue (**A**). The funnel plot from 6 studies comparing invasive ductal carcinomas (IDCs, n = 981) and normal breast tissue (n = 282) (**B**). The funnel plot from 4 studies comparing IDCs (n = 210) and DCIS (n = 458) (**C**). The funnel plot from 4 studies comparing different staged [III (n = 152) VS. I/II (n = 388)] (**D**). The funnel plot from 4 studies in determining ER-positive (n = 442) and ER-negative (n = 268) and *PTEN* hypermethylation in breast cancer patients (**E**). The funnel plot from 3 studies in determining PR-positive (n = 400) and PR-negative (n = 251) and *PTEN* hypermethylation in breast cancer patients (**F**). The funnel plot from 4 studies in determining HER2-positive (n = 263) and HER2-negative (n = 321) and *PTEN* hypermethylation in breast cancer patients (**G**).

In the current meta-analysis of *PTEN* hypermethylation and clinicopathological features, the publication biases were evaluated by the funnel plots. The publication biases were ruled out by the symmetric funnel plot (Fig. 7). The sensitivity analyses were conducted by removing one study at a time to determine the stability. The pooled ORs and HRs are not significantly changed, suggesting the stability of this meta-analyses.

## Discussion

Phosphatase and tensin homologue (PTEN) is one of the tumor suppressor which atagonizes the phosphatidylinositol 3-kinase-AKT pathway via the lipid phosphatase activity^22^. Genetic changes of *PTEN*, such as mutations and deletion, and/or epigenetic alterations are often observed in a variety of cancers^23^. In addition, PTEN functions are well known to be regulated by various posttranslational modifications including phosphorylation, unbiquitylation, acetylation, and oxidation^24^. Recent findings from Hamamoto’s group showed that SMYD2-mediated *PTEN* methylation at lysine 313 increased phosphorylation at serine 380 of PTEN, consequently inactivated PTEN and activated AKT activity and promoted cell growth and proliferation^25^. They confirmed and extended the previous notions that cross-talk of different types of posttranslational modification on histone proteins and non-histone proteins such as methylation is commonly existed^1^,^26^,^27^,^28^,^29^,^30^. Our meta-analysis demonstrated that 1). The loss of *PTEN* expression through hypermethylation plays an important role in the formation of both DCIS and IDCs, 2) *PTEN* hypermethylation does not contribute to the progression of breast cancer, 3) No correlation with *PTEN* hypermethylation was found for hormone receptor status and HER2 status.

The progression from DCIS to IDC still remains poorly understood. However it is well accepted that cancer development is arising from the accumulation of genomic changes leading to oncogene overexpression and/or loss of expression of tumor suppressor genes. On one hand, some copy number changes present in DCIS tend to increase in IDCs^31^, thus the distinct stage-specific markers have been identified in each stage^32^,^33^,^34^. On the other hand, the frequency of some copy numbers is similar to what is observed in DCIS and IDC suggesting the same cellular origin of the two components^35^,^36^,^37^. The case of *PTEN* methylation in breast cancer fits into the latter scenario, which showed no difference in *PTEN* methylation frequency in DCIS and IDC.

The results from Klajic *et al*. demonstrated that *PTEN* methylation levels were low and increased significantly in late stage III and IV^38^, however the results from the pooled study^1^,^39^,^40^,^41^ did not show the significant difference between different stage of breast cancer. Our current meta-analysis on PTEN methylation in breast cancer implied that PTEN methylation is an early tumorigenic marker for breast cancer and stays positive and stable through the whole process of malignancy from very early to advanced stage.

Only one study investigated the prognostic significance of *PTEN* hypermethylation in breast cancer. By univariate analysis they found that the high expression of PTEN which is equivalent to low hypermethylation status of *PTEN* was significantly associated with a longer relapse-free survival in patients with ER-negative an PTEN-positive status^1^. We were unable to perform quantitative analysis due to only one publication. Instead, we used 1) gene expression data and survival information of 3,557 breast cancer patients downloaded from Gene Expression Omnibus (GEO) (Affymetrix HGU133A and HGU133+2 microarrays), and 2) survival data for 995 breast invasive cancer (BRCA) patients from The Cancer Genome Atlas (TCGA), along with RNA-SEQ expression for mRNAs and miRNAs from TCGA, and lncRNA expression from MiTranscriptome beta. The online tools http://kmplot.com/breast/ and http://www.oncolnc.org were used to obtain Kaplan-Meier survival plots correspondingly. No correlation was found between low expression of PTEN and disease-free survival. However we need carefully interpret these survival data since all these data are base on mRNA expression, they may not accurately reflect the protein expression which are more crucial to gene function.

In contrast to the data from mRNA expression, a group in China recently conducted a meta-analysis to assess the association of PTEN negativity by immunohistochemistry method with overall survival and disease-free survival. They found that PTEN negativity was significantly associated with unfavourable prognosis in terms of overall survival in breast cancer although the association of PTEN negativity and disease-free survival was not validated by their study^13^.

In conclusion, the meta-analysis demonstrated that PTEN could be an early tumorigenesis marker for breast cancer, whether *PTEN* methylation with negative protein expression (not only mRNA expression) was probably associated with poor survival status needs more clinical cohort studies to confirm.

## Additional Information

**How to cite this article**: Lu, Y.-M. *et al*. The association between *phosphatase and tensin homolog* hypermethylation and patients with breast cancer, a meta-analysis and literature review. *Sci. Rep.* **6**, 32723; doi: 10.1038/srep32723 (2016).

## Figures and Tables

**Table 1:** Basic characteristics of the included studies in breast cancer.

Study	Country	Patients	Methods	Primary Aim	Methylation site
Garcia *et al*.^39^	Spain	90 BC tissue/90 normal individuals	Methylation specific-real time PCR	Determine the importance of *PTEN* silencing in sporadic BC	Promoter, CpG islands
Khan *et al*.^42^	USA	44 BC/16 normal breast tissue	MSP	Determine if differential methylation of the *PTEN* promoter region has a role in the transcriptional inactivation of the gene in invasive BC	Promoter, CpG islands
Muggerud *et al*.^43^	Norway	27 DCIS/28 IDCs/34 IDCs with DCIS/28 normal tissues	Methylation specific-real time PCR	Understand whether epigenetic changes correlate with invasive status in BC	Promoter, CpG islands
Zhao *et al*.^44^	China	92 BC/24 adjacent to BC/16 normal breast tissues	MSP	Determine the *PTEN* gene methylation in BC	Promoter, CpG islands
Klajic *et al*.^38^	Norway	199 BC/26 DCIS/17 normal breast tissues	MSP	Identify markers to understand how epigenetic changes affect BC progression	Promoter, CpG islands
Zhang *et al*.^1^	China	191 BC/normal breast tissues	MSP	Determine the methylation of PTEN and loss of PTEN expression in BC	Promoter, CpG islands
Siddiqui *et al*.^40^	India	180 BC paired with 180 adjacent normal tissues	MSP	Determine association between PTEN and clinical outcomes in BC	Promoter, CpG islands
Yari *et al*.^41^	Iran	103 BC/ 50 healthy individuals	MSP	Determine relation of PTEN methylation and risk of BC	Promoter, CpG islands

BC: breast cancer, DCIS: ductal carcinoma in situ, IDCs: invasive ductal carcinomas.
